# The Effects of Sand Particles on the Synergy of Cavitation Erosion-Corrosion of MIG Welding Stainless Steel Coating in Saline Water

**DOI:** 10.1155/2020/8876406

**Published:** 2020-09-28

**Authors:** Haodan Pan, Jun Tao, Meng E, Hongxiang Hu, Zhengbin Wang

**Affiliations:** ^1^College of Petroleum Engineering, Liaoning Shihua University, Fushun 113001, China; ^2^CAS Key Laboratory of Nuclear Materials and Safety Assessment, Institute of Metal Research, Chinese Academy of Sciences, Shenyang 110016, China; ^3^School of Materials Science and Engineering, Shenyang Aerospace University, Shenyang 110136, China

## Abstract

Cavitation erosion (CE) is a common problem troubling many flow-handling equipment such as valves, orifice plate pipes, and propellers. The coating technique is a widely used strategy to resist CE. It is important to understand the CE-corrosion behavior of the coatings in the corrosive solution, especially in the sand-containing saline water. A newly designed MIG welding precipitated hardened martensitic stainless steel (PHMSS) coating was performed, and its silt-CE was investigated in a suspension composed of 3.5 wt.% sodium chloride and 3% silica sand using an ultrasonic vibrator processor. The microstructure of the coating was characterized by optical microscopy and scanning electron microscopy. The effects of the sand particles on the CE-corrosion were analyzed using mass loss measurement, potentiodynamic polarization curve, and surface morphology observation. The results showed that the PHMSS coating was mainly composed of the lath martensitic phase alone. Its mass loss rate was in ascending order in the solution of distilled water alone, sand-containing distilled water, saline water alone, and sand-containing saline water. Sand particles played more roles in the CE in the distilled water than in the saline water. The synergy of CE and corrosion was much less in the sand-free saline than in the sand-containing saline. The maximum component was the erosion enhancement due to the corrosion in the saline without sand particles but was the pure erosion component in the saline with sand particles. The mechanism of the sand particles' effect on the CE was also discussed.

## 1. Introduction

Cavitation erosion (CE) provokes a high degree of discussion in the fields of flow-handling systems such as impellers, valves, turbines, pumps, and pipes [1–5]. It is mainly due to its complex processes including the bubble nucleation, high-pressure collapsing, and the interaction between the bubble and the material surface. Complex serving conditions also give great difficulty to the investigations including CE and corrosion. The main problem lies in the lack of understanding of the synergy between the mechanical damage caused by the CE and the corrosion caused by corroded ions. Therefore, the clarification to the synergy of the CE and corrosion is essential to the material design and equipment maintenance.

The synergy effect on the CE and corrosion has captured many results. The CE behavior in the corrosive liquid is related to the two main effects of mechanical abrasion and electrochemical corrosion [6–8]. The CE of the materials with good corrosion resistance was less affected by the corrosion-induced damage [9]. Normally, synergy components of the CE and the corrosion were higher than the sum of the pure CE and the pure corrosion [8, 10, 11], while, on some conditions, the minus synergy of the CE and corrosion is less than the pure sum-up of the CE and the corrosion [12, 13]. However, these studies above are obtained in pure liquid solutions. They are doubt to be applied to the conditions with solid particles like the impeller serving in the river with sands. Sand particles can reduce the tensile strength of the solution [14] and affect both the number of the bubble nucleation and the collapse of the bubbles [15]. Even their surface configuration and chemical composition have effects on the CE [16]. Unfortunately, few studies pay attention to the effect of the sand particles on the synergy action of the CE and corrosion. Hu and Zheng [17] investigated the effect of the sand concentration on the CE, but no synergy of the CE and corrosion was referred.

The solid particles have a complex influence on the CEs [14, 17–21]. They affect the CE in the way of changing the solution tensile strength [14], the bubble nucleation and collapse [15], and the erosion direction to the material surface [18]. The CE configurations vary with the surface morphology [16], concentration [17], and size [19, 22] of the solid particles. It can be observed that the understanding of the effect of the solid particles on the corrosion is far less than that on the mechanical damage (CE). For the passive materials, the passive film on the material surface plays an important role in the CE, especially the corrosion [23, 24]. However, the clarification of the effect of the solid particles on the corrosion remains unknown on the condition of CE.

Martensitic stainless steel attracts many investigations due to its higher resistance to the CE than the austenitic and ferritic stainless steel [25]. Metal inert gas (MIG) welding martensitic stainless steel coating is a promising method to resist the CE and corrosion, like the AISI 431 [26], AISI 410 [27], and CA-6NM [28]. Further enhancement in the resistance to the CE was also carried out on the stainless steel by the nitriding process [29, 30], laser surface melting [9], and welding [31, 32]. MIG welding is widely applied in many maintenances to the component suffering slurry erosion and CE due to its convenient operation. In the present study, a newly designed precipitated hardened martensitic stainless steel coating was prepared on 35 forged steel by a MIG welding method. Its CE-corrosion behavior was investigated in a sand suspension of 3.5 wt.% NaCl and 3 wt.% silica sand. The effects of sand particles on the interactions between the CE and the corrosion were also discussed.

## 2. Experimental

### 2.1. Coating Preparation

A newly designed wire of precipitated hardened martensitic stainless steel was used as filler wire in the MIG welding process. Its chemical composition is listed in Table 1. The MIG welding precipitated hardened martensitic stainless steel (PHMSS) coating was prepared on a cast steel, ASTM 485-275 (70-40). Before the deposition of the coatings, the substrate surface was derusted and ground to remove the surface oxides and contaminants. The MIG welding process was carried out using a Quinto-GLC403 digitalized MIG welding machine automatically with the protecting gas of argon (98%) and CO_2_ (2%).  Table 2 lists the other optimized details of the MIG welding.

### 2.2. Cavitation Erosion Test

**Figure 1:**
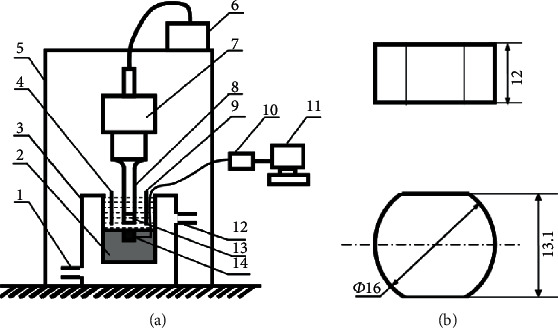
Schematic diagram of (a) ultrasonic CE equipment and (b) specimen dimensions. 1: water inlet; 2: nylon support; 3: cooling bath; 4: reference electrode; 5: soundproof enclosure; 6: ultrasonic generator; 7: transducer; 8: horn; 9: counter electrode; 10: electrochemical workstation; 11: computer; 12: water outlet; 13: horn tip; and 14: working electrode.

The CE-corrosion experiments were conducted using an ultrasonic vibratory oscillator (Q700 Sonicator) resonating at 20 kHz with a peak-to-peak amplitude of 15 *μ*m at 25 ± 1°C. The experimental processes were based on the ASTM G32-10 Standard [33]. A volume of 80 mL of different solution was used as the experimental solution in a stainless beaker including the distilled water with and without 3 wt.% silica sand (200-300 meshes) and the saline water of 3.5 wt.% NaCl with and without 3 wt.% silica sand (200-300 mesh). The test specimen was fixed in a holder as the lower specimen, which was installed coaxially with the horn tip at a constant distance of 0.5 mm. The experimental instrument and its specimen dimensions are sketched in Figure 1. All the specimens were gradually ground to 2000 grit abrasive papers, ultrasonically degreased with ethyl alcohol, and hot air-dried with a blower before the experiments. The weight of the specimens was measured by an analytical balance with an accuracy of 0.1 mg. The average value of at least three experiment data was utilized to make sure the representativeness and reproducibility.

### 2.3. Electrochemical Measurements

All electrochemical experiments were performed in the saline solution containing 3.5 wt.% NaCl at 25 ± 1°C. The experiments were carried out with an electrochemical cell (Gamry Interface 1000) accompanied by a traditional three-electrode cell comprising a standard saturated calomel electrode (SCE) as the reference electrode, a platinum slice as the counter electrode, and a sample as the working electrode [34]. The sample was prepared according to the same processes as those for the CE tests and sealed with epoxy with an exposure area of 1.83 cm^2^. Polarization measurements were conducted after 1 h open circuit potential (OCP) tests with a stable potential, and the potential range was from -0.5 V to 0.7 V vs. OCP at a scan rate of 0.5 mV/s. Each experiment was repeated at least three times to ensure reproducibility.

### 2.4. Surface Observation

X-ray diffraction (XRD, D/Max-2500PC) was employed to identify phase constituents at a Cu K*α* radiation, a voltage of 40 kV, and a current of 40 mA. Microstructures of the coatings were analyzed by optical microscopy (OM, Carl Zeiss). Before the microstructure observation, the sample surface was etched with a solution of 5 g FeCl_3_, 50 mL HCl, and 100 mL H_2_O. The microhardness of the coating was measured with an automatic microhardness tester at a load of 1.96 N and a duration time of 15 s. Stereomicroscopy (Stemi 2000, Carl Zeiss) and scanning electron microscopy (SEM, XL-30FEG) were employed to reveal the eroded morphologies after tests. The surface roughness was examined by a white light interferometer (MicroXAM).

## 3. Results and Discussion

### 3.1. Coating Characterization

**Figure 2:**
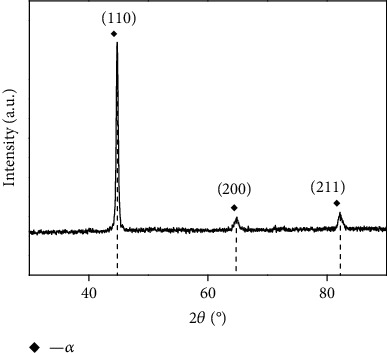
XRD pattern of the test coating.

Figure 2 shows the X-ray diffraction patterns of the coating surface. It can be seen that the diffraction peaks are located at 42°, 62°, and 81°, respectively, which are typical martensitic peaks. The coating is mainly composed of the martensitic phase with a single body-centered tetragonal (bct) structure without another second phase.

**Figure 3:**
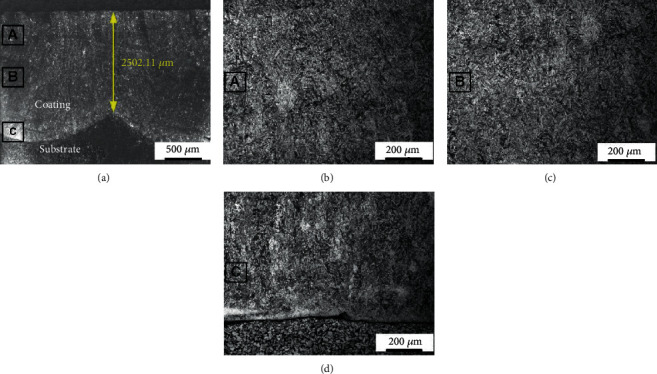
The cross-sectional metallographic images of the PHMSS coating.

Figure 3 is the cross-sectional microstructure of the stainless steel coating observed by optical microscopy. The thickness of the coating is around 2.5 mm (Figure 3(a)). Three regions are selected to reveal the details of the coating structure in the direction of thickness as labels A, B, and C. Locally magnified images are presented in Figures 3(b)–3(d) corresponding to the regions A, B, and C, respectively. A uniform structure can be observed on the cross-sectional surface of the coating. Almost no difference can be seen in the structure between the region near the top surface (Figure 3(b)), the region in the middle of the coating (Figure 3(c)), and the region near the fusion line (Figure 3(d)). A typical lath structure dominates the entire coating. It is compact with few pores, cracks, and other defects. Moreover, metallurgical bonding is formed in the interface between the coating and the substrate indicating good bonding force (Figure 3(d)).

**Figure 4:**
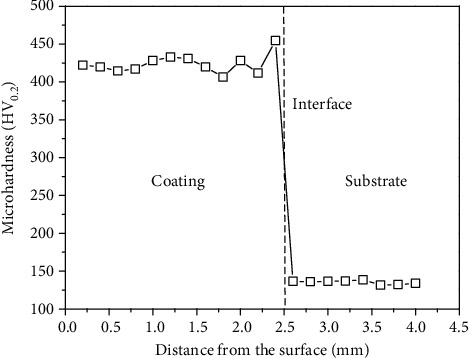
Microhardness profiles in the direction of the thickness of the MIG welding PHMSS coating and substrate.

Figure 4 displays the microhardness distribution in the direction of the thickness. The interval is fixed at 0.2 mm between the adjacent hardness points. The hardness of the coating has few fluctuations in the value suggesting a uniform structure. Its average value is 423.85 HV_0.2_, which is approximately 3.3 times that of the substrate (129.31 HV_0.2_).

### 3.2. Mass Loss

**Figure 5:**
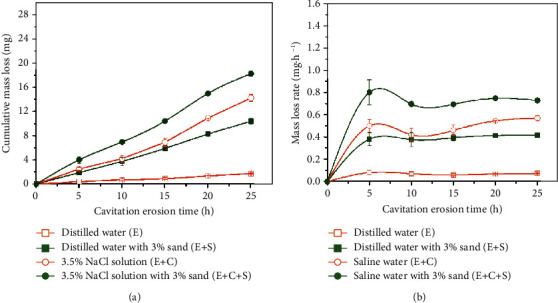
(a) Cumulative mass loss and (b) mass loss rate curves of the coating as a function of CE time in distilled water and 3.5 wt.% NaCl solution with and without 3 wt.% sand particles (200-300 mesh).

Figure 5 shows the variation of the cumulative mass loss and mass loss rate of the target coating with time in distilled water and saline water (3.5 wt.% NaCl) with and without sand (3 wt.%). The cumulative mass loss increases near linearly with test time for all the conditions (Figure 5(a)). No obvious incubation period can be found. They are 1.75 mg, 10.35 mg, 14.2 mg, and 18.2 mg after 25 h CE in distilled water, distilled water with sand, 3.5 wt.% NaCl solutions, and 3.5 wt.% NaCl solutions with 3 wt.% sand, respectively. The cumulative mass loss in the distilled water with 3% sand is nearly 6 times that in the distilled water alone. For the case of saline water with sand, it is approximately 1.3 times that in the saline water alone. Therefore, it can be deduced that adding sand particles can increase the CE in both the distilled water and the saline water. Moreover, the cumulative mass loss in the saline water is higher than that in the distilled water with and without sand particles. It indicates that chloride ions degraded the CE resistance of the coating.

**Figure 6:**
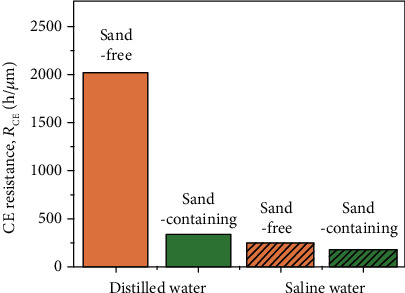
CE resistance in the distilled water and saline water with and without sand particles.

The mass loss rates' variation with time is presented in Figure 5(b). They all increase initially in the first 5 h and then stay in a relatively stable state until the end of the tests in all the solutions. However, the mass loss rate is the least in the distilled water and the most in the 3.5 wt.% NaCl solution with sand. The CE resistance (*R*_CE_) is determined based on the mean depth of the CE rate [35]. It is expressed as follows:

(1) $$\begin{array}{c} R_{\text{CE}}\left(\text{h}∙{\mu \text{m}}^{-1}\right)=\frac{1}{\text{MDER}}, \end{array}$$

(2) $$\begin{array}{c} \text{MDER}\left(\mu \text{m}\cdot {\text{h}}^{-1}\right)=\frac{\Delta m}{10\rho A\Delta t}, \end{array}$$

where MDER is the mean depth of the erosion rate, Δ*m* is the cumulative mass loss in mg, *ρ* is the density of the coating (approximately 7.7 g/cm^3^), *A* is the exposure area of the sample (1.83 cm^2^), and Δ*t* is the time interval (25 h).  Figure 6 plots the bar graph to compare the CE resistance of the stainless steel coating in different conditions. The trend is the same as that obtained by the cumulative mass loss (Figure 5(a)). It can be conducted that the CE resistance in the distilled water is approximately 5.9 times that in the sand-containing distilled water, whereas in the sand-containing saline water, it is about 1.3 times that in the sand-free saline water. It is reasonable to say that sand particles play a negative role in alleviating the CE damage in both distilled water and saline solution. Furthermore, they have a greater effect on the distilled water than on the saline water. It might be because the chloride ions reduced the influence of the sand particles on the CE.

### 3.3. Electrochemical Behavior

**Figure 7:**
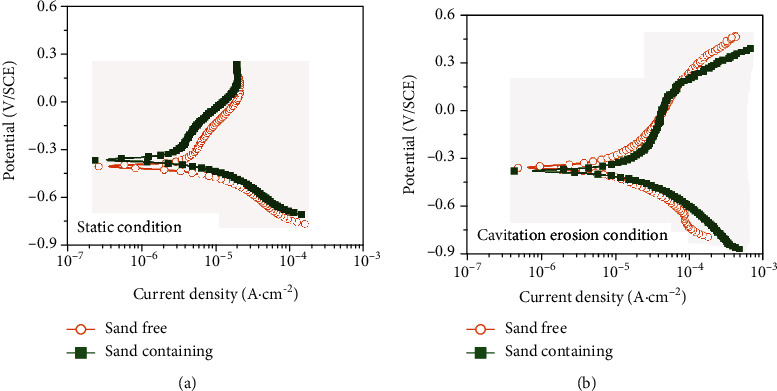
Potentiodynamic polarization curves of the PHMSS coating under (a) static and (b) CE conditions in 3.5 wt.% NaCl solution with and without sand particles.

Figure 7 reveals the potentiodynamic polarization curves of the PHMSS coating on the static and CE conditions with and without sand.  Table 3 lists the corrosion current density (*I*_corr_) and corrosion potential (*E*_corr_) obtained from the Tafel fitting. Under the static condition, the corrosion potential shifts positively by 42.8 mV, and the *I*_corr_ decreases by 1.7 *μ*A/cm^2^ in the NaCl solution with sand particles compared with the case without sand particles (Figure 7(a)). It indicates that the existence of sand particles can slightly alleviate the corrosion of the PHMSS coating. Moreover, the anodic current density is decreased more obviously in the sand-containing NaCl solution than that in the sand-free NaCl solution on the static condition. It may be caused by the absorption of the fine sand particles to the sample surface, which can isolate the part of the corrosive-reducing anode. However, the corrosion behavior differences are small on a whole between the sand-free saline solution and the sand-containing saline solution. Moreover, passivation can be observed on the static conditions with and without sands, and the passivation current densities are the same in an order of magnitude of approximately 2.0 × 10^−5^ A/cm^2^.

On the CE conditions, the corrosion tendency changes opposite to that in the static conditions with sands (Figure 7(b)). The *I*_corr_ increases by approximately 6 times after adding sands. The corrosion potential moves negatively from -167.2 mV_SCE_ in the sand-free solution to -251.8 mV_SCE_ in the sand-containing solution. It can be deduced that sand particles accelerate the corrosion process. Besides, the PHMSS coating shows no passivation phenomenon on the CE conditions neither with nor without sand particles. It exhibits an active corrosion status in both CE conditions. Compared with the static condition, the corrosion current density is relatively high on the CE condition independent on the sand particles. This is mainly due to both the mechanical damage to the passive film and the accelerated mass transfer during CE, which promotes the corrosion of PHMSS coating.

### 3.4. Morphology

CE can cause two different erosion regions: the center region and the perimeter region [36]. This can be changed by adding sand particles depending on the sand concentration [17], where the boundary between the center region and the perimeter almost disappeared at the concentration of 3 wt.% sand particles. The optical stereomicroscopy was used to reveal the macromorphologies after CE in different solutions.

**Figure 8:**
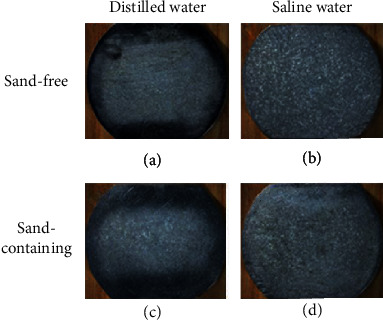
Macrographs of the PHMSS coating surface after CE for 25 h in (a) sand-free distilled water, (b) saline water, (c) sand-containing distilled water, and (d) sand-containing saline water.

Figure 8 shows the representative macromorphology of the eroded surface after CSE for 25 h in distilled and saline water with and without sand. It can be observed that the two regions can be distinguished in the distilled water (Figures 8(a) and 8(c)), but it is not as obvious as that caused by different sand particles [17], while the boundary between the center region and the perimeter region cannot be observed in the saline water conditions. Moreover, the CE is more severe in the saline water than in the distilled water, indicating the acceleration of the corrosion. To get an insight into the material removal mechanism, SEM observations were carried out on both the perimeter and center regions.

**Figure 9:**
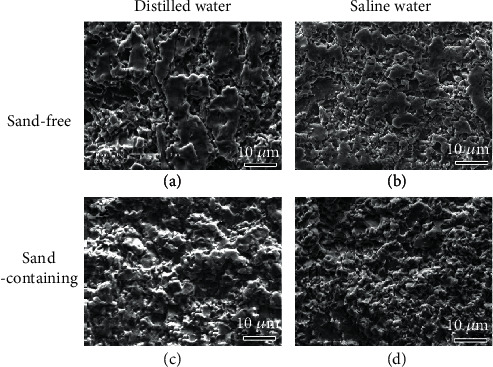
SEM micrographs of the CE surface in the central region after CE for 25 h in (a, c) distilled water and (b, d) 3.5 wt.% NaCl solutions (a, b) without and (c, d) with sand.

Figure 9 displays the CE surface morphologies in the center region of the sample after the test for 25 h in the distilled water and saline water with and without sand particles. The surface remains the original surface locally after the test in sand-free distilled water (Figure 9(a)). It indicates that the stainless steel has good CE resistance in the distilled water without sand. In contrast, the erosion is much more severe in the saline water than in the distilled water after the same test time (Figure 9(b)). Only small parts of the original surface can be observed as well as more CE craters. Without sand particles, the degradation of the coating resistance to CE is mainly due to the chloride ions.

Compared with the case in the sand-free solution, the CE is accelerated in both distilled water and saline water after adding the sand particles (Figures 9(c) and 9(d)). All the original surfaces have been removed. However, the morphology for the sand-containing distilled water (Figure 9(c)) is different from that for the saline water (Figure 9(d)), indicating different damage mechanisms. The density of the crater for the former case is less than that for the latter case. It seems that the surface in Figure 9(d) was obtained by the surface in Figure 9(c) after further CE. No big particle-like extrusion can be found on the surface tested in the sand-containing saline (Figure 9(d)). Anode dissolution preferentially occurs on the sharp corners and edges caused by the mechanical cutting of silt-CE resulting in further refinement to the erosion surface. Moreover, the pitting can also increase the bubble nucleation and accelerate the CE. Therefore, the eroded surface in the sand-containing saline is worse than that in the saline water and distilled water.

**Figure 10:**
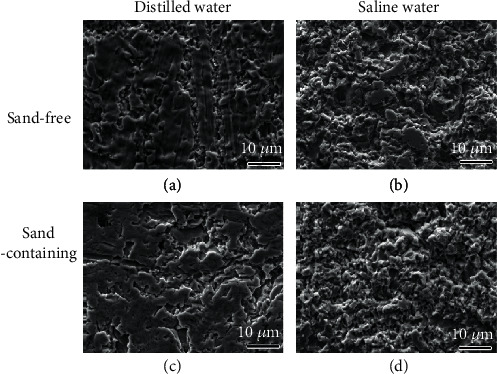
SEM micrographs of the CE surface in the perimeter region after CE for 25 h in (a, c) distilled water and (b, d) 3.5 wt.% NaCl solutions (a, b) without and (c, d) with sand.

Micrographs observed by SEM in the perimeter region of the PHMSS coating surface after the CE test in different conditions are shown in Figure 10. The damage in the perimeter region follows the same trend as that in the center region. It is more severe in the sand-containing solution. On the condition of distilled water alone (Figure 10(a)), the original surface can still be seen even after the 25 h test. It only suffers slight plastic deformation due to the microjet flow or shock wave, and the damage degree is relatively low to that in the center region (Figure 9(a)). After adding the sand particles, the erosion regions are enlarged (Figure 10(c)). Local isolated craters connect each other and become bigger craters. Particle impingent due to the collapsing bubbles brings extra mechanical erosion to the coating surface [17]. In the saline solution without sand particles (Figure 10(b)), the original surface can be also observed but with less area than that in the distilled water alone, while no original surface can be found after the same CE time in the solution with sand particles (Figure 10(d)). It indicates that the sand particles affect the CE process in the entire surface of the sample, which is consistent with that observed in the macromorphology observations (Figure 8(d)).

Compared with the CE in the center region (Figure 9), the CE degree is relatively low in the perimeter region in the distilled water but similar in the saline water. It means that the sand particles' effect is more obvious in the distilled water than in the saline water, which well agrees with the result obtained from *R*_CE_ (Figure 6).

**Figure 11:**
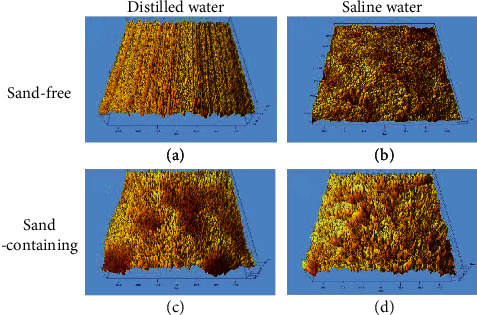
Three-dimensional roughness of the center region of the PHMSS coating surface after CE for 25 h in distilled water and saline water with and without sand particles.

Figure 11 displays the roughness profiles of the PHMSS coating surface after CE for 25 h in different solutions. All the areas measured for the roughness are selected within the center region of the surface. In the sand-free distilled water, the eroded surface is uniform and the grinding trace can still be seen (Figure 11(a)), which is consistent with that observed by SEM (Figure 9(d)). No obvious CE craters are detected suggesting little mass loss, which well agrees with the cumulative mass loss in Figure 5(a). Therefore, it can be deduced that the coating resistance to the CE is good in the distilled water alone in the view of roughness. In contrast, the grinding trace is completely removed after the same test time in the distilled water containing sand particles (Figure 11(c)). Local craters exist indicating the sand particles accelerated the CE, which well agrees with the mass loss measurements (Figure 5(a)), while the variations in the roughness morphologies in the saline water are not as big as those in the distilled water. However, the surface is coarser in the sand-containing saline than in the sand-free saline. The size of the peaks and the craters is larger than that in the sand-free saline.

**Figure 12:**
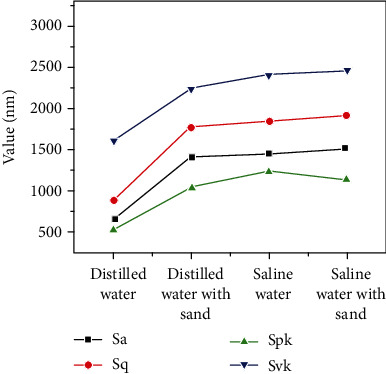
Relationship between the surface roughness and the solution conditions after 25 h CE.

The quantitative comparison of the surface roughness features after CE for 25 h in different solutions is showed in Figure 12. Sa and Sq are the arithmetical mean height and root mean square height, respectively. Spk is the reduced peak height representing the mean height of peaks above the core surface; Svk expresses the arithmetical mean of the reduced valley depth of the areal material ratio curve. Essentially, this is a measure of the valley depth below the core roughness. It can be observed that all the parameters increase significantly after adding sand to the distilled water. They change in the same way with the changing solution. The values of Sa, Sq, Spk, and Svk slightly rise in the solution adding NaCl alone and the solution adding NaCl+sand. It suggests that the maximum value of the peaks and the valleys increased due to the combined action of the particle impingement and CE. Sa in the sand-containing saline water is approximately 2.3 times that in the distilled water alone and approximately 1.04 times that in the saline water alone. It indicates that the sand particles degrade the CE resistance of the MIG PHMSS coating more in the distilled water than in the saline water. This may be mainly own to the increase in mechanical damage.

### 3.5. Synergy Action between CE and Corrosion

It is necessary to understand not only the quantity of synergistic effects [22] but also the sand particles' effect on the synergy. The synergy components were obtained in the solution with and without sand particles, respectively. The total damage of CE can be described in the way of volume loss by the following equations [37–39]:

(3) $$\begin{array}{c} V_{\text{T}1}=V_{\text{E}}+V_{\text{C}}+V_{\text{S}1}=V_{\text{E}}+V_{\text{C}}+V_{\text{EIC}}+V_{\text{CIE}}, \end{array}$$

(4) $$\begin{array}{c} V_{\text{T}2}=V_{\text{E}}+S+V_{\text{C}}+V_{\text{S}2}=V_{\text{E}}+S+V_{\text{C}}+V_{\text{ESIC}}+V_{\text{CIES}}, \end{array}$$

where *V*_T1_ and *V*_T2_ are the total volume loss of the coating in the saline solution without and with sand particles, respectively; *V*_E_ and *V*_E+S_ are the pure mechanical erosion components caused by the CE alone in the distilled water without and with sand particles, respectively; *V*_C_ is the pure corrosion component measured in the saline water; *V*_S1_ and *V*_S2_ are the synergy components of the CE and corrosion in the saline water without and with sand particles, respectively; *V*_EIC_ and *V*_ESIC_ are the corrosion enhancements induced by the corrosion in the sand-free and sand-containing saline solutions, respectively; and *V*_CIE_ and *V*_CIES_ are the erosion enhancements induced by the corrosion in the sand-free and sand-containing saline solutions, respectively. In the present work, *V*_E_ and *V*_T1_ were measured after CE for 25 h in distilled water and saline water without sand particles, respectively. *V*_E+S_ and *V*_T2_ were obtained by the same method but in the sand-containing solution. *V*_C_ was calculated from the *I*_corr_ from the polarization curve in the static saline solution according to Faraday's law. *V*_EIC_ and *V*_ESIC_ were determined by the same method but on the CE condition in the saline water without and with sand particles, respectively. It is noted that they are the pure enhancement components due to erosion, which means that the pure corrosion component (*V*_C_) has been deducted.

**Figure 13:**
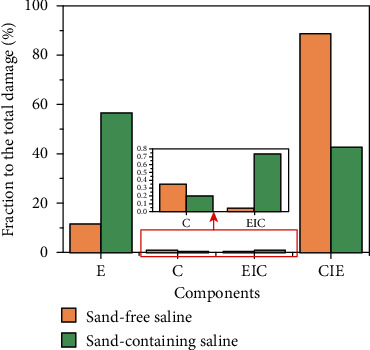
Fraction of each component to the total damage.

Table 4 lists the components described above for the MIG welding HPMSS coating. On the sand-free conditions, the biggest component is the erosion enhancement (*V*_CIE_) caused by the corrosion with a fraction of 88.14% to the total damage. The next one is the pure CE (*V*_E_, 8.89*E*-3 mm^3^/h), and the minimum one is the corrosion enhancement due to CE only at the fraction of 0.05%. Compared with the sand-free conditions, a great change occurs due to the existence of the sand particles. Pure mechanical erosion caused by the CE (*V*_E+S_) becomes the dominant component followed the erosion enhancement due to the corrosion (*V*_CIES_). The pure corrosion component turns out to be the minimum one. All these changes result in the increase of the total damage by approximately 19% compared with that in the sand-free saline water.  Figure 13 plots the bar graphs of the fraction of each component to the total damage. It can be also observed that the biggest component transforms from *V*_CIE_ to *V*_E+S_ due to the adding of the sand particles. The synergy in the sand-free saline is much less than that in the sand-containing saline. Moreover, the corrosion-related components (*V*_C_ and *V*_EIC_) are significantly less than the erosion-related components (*V*_E_ and *V*_CIE_) indicating the good corrosion resistance of the coating.

The effect of solid-phase loading on the erosion-corrosion behavior for stainless steel has been examined. It was reported that the increase of charge transfer resulted in a higher contribution to the total weight due to corrosion-related effects [40]. However, the foundation in the present work is different. With the addition of the sand particles, the corrosion-related effect is reduced and the pure mechanical erosion is enhanced. Two aspects may contribute to the decrease of the corrosion effect. One is due to the good corrosion resistance of the MIG welding PHMSS coating itself. The other can result from the sand particles partially preventing the diffusion of chloride into the coating surface. It proved the decrease of the corrosion component (from *V*_C1_ to *V*_C2_) after adding sand particles.

The sand particles affect the silt-CE in many aspects including corrosion, erosion, and synergy. The total damage is increased by adding the sand particles in both the distilled water and the saline water (Figure 5). It is the sum results of each damaged component. On the static distilled water, pure CE is dominant but limited, which can be proved by the small mass loss (Figure 5). Microjet and shock wave do not tear the coating surface (Figures 9(a), 10(a), and 11(a)), while all these are changed after adding sand particles to the distilled water. The combined action of the sand particle erosion and CE breaks the integrity of the coating resulting in great material removal. In addition to the case in the distilled water, more complex processes occur in the silt-CE and corrosion. The passivation film is peeled off leading to an increase of the *I*_corr_. That is why the corrosion enhancement due to erosion becomes higher than that in the sand-free saline water (Table 4). Moreover, the variation of the corrosion component resulted from not only the change of the surface area but also the disturbance of local mass transfer and the concentration due to the moving into and out of the boundary layer. A great change in the pure mechanical erosion occurs when adding the sand particles in the saline water (Table 4), which results in the degradation of the surface morphology (Figures 9(b), 10(b), and 11). It indicates that sand particles play an important role in the mechanical erosion to the coating surface. According to the report carried out by Hu and Zheng [17], the silt-CE was mainly determined by sand erosion on the sand concentration of 3 wt.%. Therefore, it is easy to understand why the pure erosion component becomes the dominant one to the other components.

## 4. Conclusions

A MIG welding HPMSS coating was newly designed on the cast steel. Its silt-CE resistance was investigated through the mass loss measurement and the micromorphology observation. The synergy of CE and corrosion was discussed in the 3.5 wt.% NaCl solutions without and with sand particles. Some conclusions can be drawn as follows:

1. The MIG welding PHMSS coating is mainly composed of the lath martensitic phase alone. It has a single body-centered tetragonal (bct) structure with the microhardness of 423.85 HV_0.2_.
2. The mass loss rate of the coating is in ascending order in the solution of distilled water alone, sand-containing distilled water, saline water alone, and sand-containing saline water.
3. The synergy in the sand-free saline is much less than that in the sand-containing saline. The maximum component is the erosion enhancement due to the corrosion in the saline without sand particles but is the pure erosion component in the saline with sand particles.

## Figures and Tables

**Table 1:** Chemical compositions of the welding wire (wt.%).

Comments	C	Cr	Ni	Mo	Nb	Cu	Si	Mn	P	S	Fe
Content	0.024	16.30	4.92	0.40	0.21	3.52	0.45	0.61	0.012	0.03	Bal.

**Table 2:** Process parameters of the MIG surfacing.

Process parameters	Value
Wire speed (m∙min^−1^)	12.00
Pulse frequency (Hz)	220
Base current (A)	140
Pulse time (ms)	2.20
Pulse voltage (V)	37
Pulse shape	Steep

**Table 3:** Comparative summary of electrochemical corrosion results for the PHMSS coating in 3.5 wt.% NaCl solution with and without 3 wt.% sand particles.

Conditions	*I*_corr_ (*μ*A/cm^2^)	*E*_corr_ (mV vs. SCE)
Sand-free (static)	3.026	-273.6
Sand-containing (static)	1.326	-153.1
Sand-free (CE)	0.582	-167.2
Sand-containing (CE)	3.538	-251.8

**Table 4:** Component values calculated after 25 h CE in the solution without and with sand.

Conditions	Volume loss rates (10^−3^mm^3^/h)/fraction				
Sand-free	*V*_T1_	*V*_E_	*V*_C1_	*V*_EIC_	*V*_CIE_
	77.51	8.89	0.26	0.04	68.32
Sand-containing	*V*_T2_	*V*_E+S_	*V*_C2_	*V*_ESIC_	*V*_CIES_
	92.50	52.60	0.18	0.68	39.04

## Data Availability

The data of mass loss, electrochemistry, and hardness used to support the findings of this study are included within the article.
